# Electrospun Nanofibers for Label-Free Sensor Applications

**DOI:** 10.3390/s19163587

**Published:** 2019-08-17

**Authors:** Nahal Aliheidari, Nojan Aliahmad, Mangilal Agarwal, Hamid Dalir

**Affiliations:** 1 School of Mechanical Engineering, Purdue University, West Lafayette, IN 47907, USA; 2 Integrated Nanosystems Development Institute (INDI), Indiana University-Purdue University Indianapolis, Indianapolis, IN 46202, USA; 3 School of Electrical and Computer Engineering, Purdue University, West Lafayette, IN 47907, USA; 4 Purdue School of Engineering and Technology, Indiana University-Purdue University, Indianapolis, IN 46202, USA

**Keywords:** label-free sensors, biosensor, electrospinning, nanofibers

## Abstract

Electrospinning is a simple, low-cost and versatile method for fabricating submicron and nano size fibers. Due to their large surface area, high aspect ratio and porous structure, electrospun nanofibers can be employed in wide range of applications. Biomedical, environmental, protective clothing and sensors are just few. The latter has attracted a great deal of attention, because for biosensor application, nanofibers have several advantages over traditional sensors, including a high surface-to-volume ratio and ease of functionalization. This review provides a short overview of several electrospun nanofibers applications, with an emphasis on biosensor applications. With respect to this area, focus is placed on label-free sensors, pertaining to both recent advances and fundamental research. Here, label-free sensor properties of sensitivity, selectivity, and detection are critically evaluated. Current challenges in this area and prospective future work is also discussed.

## 1. Introduction

Among all the spinning methods that can be used to fabricate micro- and nanofibers, including melt spinning, solution spinning and emulsion spinning [1], electrospinning is widely regarded as the best method to achieve continuous and uniform fibers on the nano and micro scale. In this process, filament development is based on the uniaxial stretching of a material from a feeding jet in the presence of an electric field. This process aids in creating uniformity and stability, with no disruption of the continuous electrospun fiber [2].

In this process, a viscoelastic solution (typically polymer-based) is needed. Here, diverse types of polymers and solvents have been used to develop different fiber structures, pore size and shape. Parameters such as viscosity, elasticity, and surface tension of the spanned solution can be adjusted through varying polymer and solvent ratios. In addition, molten polymers have also been used to create solvent free fibers [3]. Importantly, the degradability and biocompatibility of the polymers used must be considered in specific applications such as biomedical applications for spanned fibers [4]. 

**Figure 1:**
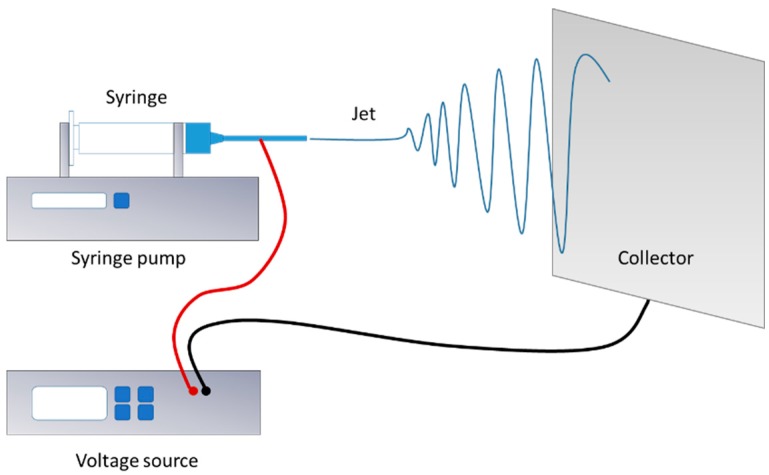
Schematic of an electrospinning setup.

In the electrospinning setup shown below, Figure 1, the polymer solution is placed in a syringe, and attached to a needle in order to create a jet. Electric voltage is applied between the needle and the collector. When the solution is ejected from the tip of the needle, the applied voltage induces charge inside the fluid, thereby inducing a Taylor cone formation. This results in the formation of a filament, which then travels from the needle tip to the collector.

To date, over 100 polymers have been successfully adopted for electrospinning. Of these, polymers such as polyurethane [5], polycarbonate [6], polyacrylonitrile [7], polyvinyl alcohol [8], polylactic acid [9], polymethacrylate [10], polyethylene oxide [11], polyaniline [12], polyethylene terephthalate [13], polyamide [14], and polyvinylchloride [15] have become the most common.

Aside from polymer type, there are many additional parameters that can affect the resulting fiber’s properties, such as altering its morphology from a beaded to a porous fiber [4]. Table 1 summarizes the effect of different ambient and solution conditions on filament formation. 

These parameters are related to either the solution or the electrospinning setup itself. The main solution parameters that have a high influence in the final properties of the fibers are polymer molecular weight, viscosity, polymer chain entanglements, solution concentration, surface tension, conductivity, dielectric effect and the solvent used. Here the parameters can be adjusted accordingly while most of the ambient or solution conditions might be different for each polymer. Table 2 shows the desired viscosity for making uniform fibers via electrospinning using different polymers.

The reports are not limited to the cases presented in Table 2; working with poly ethylene oxide (PEO) has revealed that the optimum viscosity to fabricate the electrospun nanofibers is 800–4000 cps [25]. In another study, with poly(vinylpyrrolidone) ethanolic solution electrospun fibers, it was reported that for solutions with low viscosity (i.e., below 123 cps), the structures changed to bead form [26]. The viscosity is a relative property to many parameters such as type and molecular weight of polymer or solvent, the optimum value of viscosity is defined by uniform nanofiber formation. Moreover, the molecular weight of the polymer can impact viscosity and change the morphology of the fabricated fibers [27].

Furthermore, electrospinning setup parameters such as voltage, flowrate of the solution, needle size, distance, temperature and the shape, size, and type of the collector can also affect the properties of the spanned fibers [3,4,28]. For example, with respect to collector type, having a fixed collector will result in the formation of randomly oriented filaments whereas rotating drums can generate more aligned fibers. Due to the impact of all of these parameters, a very precise setup and solution is required to achieve fibers with the desired properties, from thickness and length to porosity and surface roughness [29].

### Electrospining Methods Development

Conventional electrospinning started by using a needle like nozzle to fabricate fibers. Single nozzle solution electrospinning is the most common conventional electrospinning technique [30]. This method is the primary technique used to make filaments with different thicknesses. The solution is extruded using a single nozzle, so it is only suitable for single solutions. Therefore, a single viscous solution of polymer combined with other desired materials is needed to make filaments. While this method is inexpensive and easy to use, there are several restrictive factors. One key limitation is that it cannot be used with non-spinnable solutions. To overcome this limit, a new method called coaxial electrospinning has been developed. Coaxial electrospinning enables the simultaneous spinning of multiple different solutions [31]. Here, a double layer nozzle is made with a larger outer capillary and a smaller inner capillary. This is used to form a smaller precursor and a larger shell fiber surround [32]. This method can be used to spin non-spinnable fibers or two different polymer-based solutions to make complex fibers [33,34]. 

Aside from coaxial spinning, a side-by-side method has also been developed to be used with multiple solutions. This method reduces the complexity of coaxial spinning. Here, a side-by-side nozzle is developed with two separated capillary chambers, to extrude a blend of different polymers with different properties [35]. Another alternative method is melt electrospinning. This method is only applicable to thermoplastics, but it can eliminate the need for adding solvent. In this method, heating the nozzle (via heat gun or heating element) lowers the viscosity of the polymer to the point where it becomes spinnable. This method can also be used in with samples that are sensitive to solvents and create fibers that are stabilized at room temperature [35,36]. In certain cases, while a solution many not be highly viscous, electric charges may still inhibit the solution from stretching and being spinnable. For such cases, gas jacket spinning has been developed. In this method, a heated gas jacket is created around the spinning solution. This channel of hot air can form smaller fibers with diameters in nanometer range, while the hot air vortex controls the deposition of fibers significantly [37].

While there are multiple methods of electrospinning, there is a critical need to develop large-scale electrospinning strategies for commercial applications. Multiple jet electrospinning is one of the best configurations to deposit multiple materials or cover larger areas. This technique uses multiple nozzles to spin over larger areas and/or to deposit different layers on top of each other, following respective drying steps [38]. Centrifugal force electrospinning is another method that can produce well-controlled nano-sized fibers with proper alignment in larger scales. Here, a rotary collector is used to spin fibers using centrifugal force [39]. Building on this, needle-less electrospinning has also been introduced to eliminate the need for sophisticated nozzles and pumping stations in order to further reduce the complexity of making fibers through electrospinning. In this method, the nozzle is replaced by a charged conical or a drum shape spinner that rotates over the collector. By applying an electric force, the solution over the drum is stretched between the drum and the collector to form fibers [40]. 

Electrospinning is a simple, functional and low-cost method to produce the nanofibers [41]. Combination of advanced material science and conventional methods open a new horizon to fabricate a fully functional structures which can employed in many fields from biomedical to electrical applications [42]. In this review, we have briefly discussed applications of the electrospun materials with emphasizing on biosensors and its categories. With respect to this area, focus is placed on label-free sensors, pertaining to both recent advances and fundamental research. We highlight the latest literature on label-free sensors studies and their detection methods. Finally, the method limitations, challenges, and prospective trends are discussed.

## 2. Electrospinning Applications

**Figure 2:**
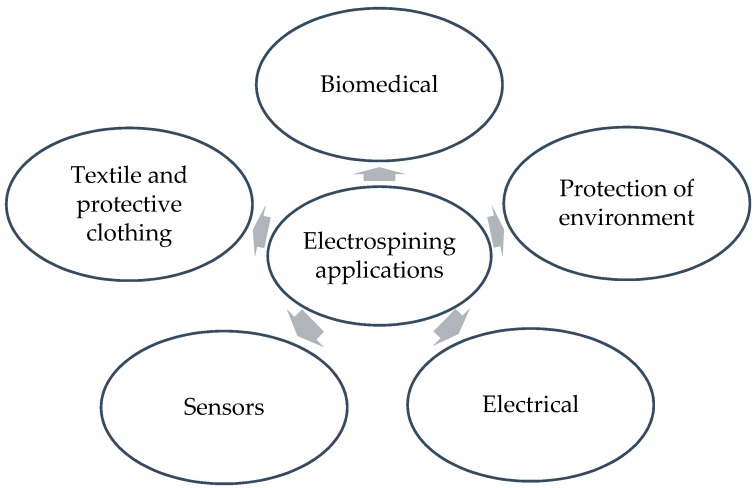
Electrospun nanofiber applications.

With outstanding surface-to-volume ratio, high porosity, enhanced physicomechanical properties, and the ability to tailoring structure and composition, electrospun nanofibers have the potential to greatly advance a variety of fields. Electrospinning has gained a great deal of attention not only because of its versatility in making micro and nano-sized fibers, but also its ability to fabricate 0,1,2 and 3D structures [43,44]. Control over size and morphology results in diverse functionalities, thereby making fibers applicable to a wide array of applications. Figure 2 shows several popular applications of electrospun nanofibers; importantly, applications are not limited to these examples. As there are numerous reviews on nanofiber applications [3,45,46,47,48,49,50,51,52,53], a few popular applications with main focus on biosensors have been introduced. 

### 2.1. Biomedical Applications

Drug delivery, wound healing, tissue and bone engineering represent well-known applications of electrospun nanofibers within the biomedical field [28,54,55,56]. Electrospun fibers have the ability to copy the hierarchical architecture of an extracellular matrix (ECM) and have therefore been extensively researched for tissue and bone engineering applications [57]. In other words, electrospun fibers, which follow the scaffold structure, can be used as biodegradable scaffolds. This scaffold is a temporary part that allows cell seeding and proliferation [42]. In a study by Xue et al., a special class of scaffold with nanofibers ordered in a radial direction was shown to enhance regeneration and wound healing capabilities. Its radial orientation directs dural fibroblasts towards the center of the wound [57]. Further research has also been done on wound dressing materials, where electrospun nanofibers have been coated with nanoparticles in order to restriction micro-organism respiration, which in turn limited micro-organism growth. These wound dressing materials, produced by electrospinning, also showed a good water holding capabilities which is essential to promote healing [47].

Electrospinning can also be helpful in fabrication of biodegradable and biocompatible membranes for tissue engineering. Different biocompatible polymers such as polylactic acid, or poly lactic-co-glycolic acid materials have been used to make bio-friendly and highly porous membranes through electrospinning [58]. In addition, a high strain gelatin-based membrane has also been developed for bone tissue engineering, where this biocompatible membrane can provide the high mechanical properties and stability needed for orthopedic applications [59]. 

Other studies have focused on the use of nanofibers in drug delivery. Recently, studies on the delivery pattern of an antibiotic drug loaded into nanofibrous mats were carried out in vitro. It was shown that the drug was released completely over the time frame of 48 h. Moreover, a device for safe and effective cancer treatment based on nanofibers material was also recently developed. It has been used as an implant mat that showed high efficiency in destroying tumor cells, with a low amount of drug loading and drug administration frequency. However, despite these advantages, there are limitations in crossing blood vessels, limited solubility and nonspecific uptake of the drug [47,60]. Recently, electrospun polymer nanofibers have also been proposed for soft tissue prosthetic applications including blood vessels, vascular, breast etc. [3].

In addition, theranostic systems have also been developed through electrospinning processes to encapsulate targets, drugs, and even diagnosing agents. Here, a double extrusion technique has been applied to create a double-layered coaxial filament. The core of these filaments can be loaded while the polymer shell layer can be functionalized to provide targeted delivery [61]. Materials such as Eudragit E100, ketoprofen, gelatin and zein are some of the best candidates for these coaxial fibers. The casing of the fibers are mostly dissolvable at certain pH levels or in specific solvent conditions, thus drugs or targets can be released more efficiently and constantly [62,63,64,65,66]. 

### 2.2. Environmental Protection

Polymeric nanofibers have been used in air filtration systems for a long time. Structural elements and channels attained by electrospinning could be at the same range of pollutant particles allowing the fibers capable to capture them. Polymer nanofibers can also be electrostatically charged and modified to enhance electrostatic attraction of particles without any additional flow resistance [42]. Different highly porous membranes have been made to absorb particles from air. Furthermore, coating these fibers with antibacterial materials can also lead to develop antibacterial membranes [67,68]. 

Electrospun nanofibers can also be used to remove volatile organic compounds (VOC) [51] and because of their high surface ratio, they are excellent candidates for liquid filtration. By using a porous structure and specific polymers, resultant fibers can be employed as a membrane or absorbent for many applications such as oil spill clean-up [50]. 

### 2.3. Electrical Applications

High conductivity, large surface area and structural stability make carbon nanofibers suitable for applications in electrical engineering and electrochemical energy storage [69]. A good number of researches has and continues to be conducted on electrospun fibers for energy storage devices such as different types of batteries, fuel cells and supercapacitors [70,71,72,73]. Polymeric nanofibers have been employed as cathode, anode and separator materials in batteries. Furthermore, these fibers also have an application as a gel electrolyte, after absorbing the electrolyte solution, thanks to their high porosity.

Conductive membranes made using nanofibers also have potential to applications in electrostatic dissipation, corrosion protection, electromagnetic interference shielding and photovoltaic devices [74,75,76]. For example, an optical shutter device based on electrospun nanofibers has been reported for a liquid crystal material that is covered with a layer of nanofibers. An electric field allows the regulation of transmissivity of the liquid crystal/nanofiber composite [3].

### 2.4. Textile and Protective Clothing

As its name indicates, protective clothing consists of textile structures designed to protect the body from degraded organic compounds such as dyes, pesticides, chemical warfare stimulants, and much more [47,77]. Their high specific area, thanks to the porous structure of electrospun fibers, makes them great materials for protective clothing, as properties such as comfort, breathability, weight, barrier properties and water vapor permeability are considered when evaluating the performance of clothing [77,78].

Electrospinning has also been applied to manufacturing of smart textile structures that can respond to external stimuli such as thermal, mechanical and electrical changes [79,80]. In a recent study, paraffin wax and polyacrylonitrile solutions were used to fabricate a core–sheath structure through a coaxial electrospinning technology. The method represented an effective way to overcome paraffin wax leakage. The smart textile was successfully tested for thermo-regulation and showed very good stability even after 500 cycles [81]. In fact, coaxial electrospinning is becoming increasingly popular in smart textiles. In another study by Yi et al., polyvinyl butyral (PVB) in ethanol was used as a sheath solution while octadecane was employed as a core. This research showed that the PVB solution and core feed rate had a great impact on filament morphology. The results revealed a high latent heat for smart textiles and good stability up to 100 cycles [82].

Bringing functionality to fibers raises the possibility of employing these substrates directly as wearable devices. Piezoelectric smart fabrics from polyvinylidene fluoride-co-trifluoroethylene (PVDF-TrFE) have been produced using an electrospinning process and the effect of post processing (e.g., thermal annealing) and material properties were investigated. Here, researchers claimed that post processing improved the mechanical properties of electrospun twisted yarn by enhancing the degree of crystallinity, increasing the strength and elastic modulus, as well as enhancing the alignment of polymer chains. These improvements made the final product very suitable for energy harvesting applications [79].

### 2.5. Sensors

Comparing all the many methods that have been employed to fabricate sensors, including compression molding, solution casting, microfabrication and more, electrospinning has become widely used and well developed within this field. Exceptional properties, such as fiber continuity, surface functionality, mechanical performance and large surface to volume ratio make this method enticing. With these properties, resulting electrospun sensors show high sensitivity, good recovery, and great sensory response.

Gas, biological substances, electrochemical, optical and thermal sensors are some previous examples applications that have utilized electrospinning. Acoustic wave, resistive and photoelectric sensors have been successfully adopted for gas sensing [83]. Furthermore, electrospun fibers have been recognized as a functional platform for immobilizing biological molecules [53]. Interestingly, incorporating nanomaterials increase immobilization due to the high surface area and free energy. In this case, biomolecules attach easier to nanomaterials resulting in higher adsorption higher and activity compared to bulk materials [46,53].

Different categories can be selected to define sensor division. In this review, sensors have been divided to two main categories: label and label free sensors, which are distinguished below.

## 3. Label-Based and Label-Free Biosensors

Biosensors refer to analytical devices that involve biological sensing elements [84]. They are capable of transforming biological response into an electric signal. They include two major components: biorecognition elements (antibody, nucleic acids, enzymes, whole cells, peptides, lectins or glycans), which recognize the target, and physico-chemical transducers, for converting the recognition event into an assessable signal proportional to the analyte target concentration [85,86,87].

Biosensors have been established for many different analytes, with different size ranges varying from small molecules to whole viruses and bacteria [88]. Due to their high sensitivity, specificity, and real-time analysis capability, together with their fast response time and low cost, biosensors have attracted attention in various fields, including food and water monitoring, clinical diagnostics, industrial and environmental monitoring, etc. [89]. New ways for synthesizing and fabricating advanced materials such as novel biorecognition elements, functional polymers, and nanofibers that can be employed as interfacial or transducer features are of critical importance [90,91,92].

The first concept for a biosensor was proposed by Clark in 1962 for glucose detection via the electrochemical detection of oxygen or hydrogen peroxide [93,94]. That was the first “enzyme-electrode”, and the first analyzer for measuring glucose in blood [95]. During the next decade, biosensors turned into a hot topic and developed rapidly. Second-generation biosensors were introduced soon after and were based on redox mediators [96]; later, a third generation of direct electron transfer (DET) sensors was made known in the 1990s [97]. Since then, a revolution has happened in the field of biosensors. In 1985, only about 30 papers were published by Elsevier, but that number shot up to 4500 in 2012 [98]. Over the years, researchers have worked to effectively enhance the detection limits, sensitivity and selectivity of biosensors to the point were glucose biosensors, such as those originally proposed by Clark, are now widely used in hospitals and clinics.

Biosensors can be categorized in different ways, including signal transduction and bioreceptor types, as well as affinity-based biosensors. In transducer mechanisms, the biological element reacts with a target analyte, and a signal will be produced through a transducer. Generally, the transducer is an analytical tool that provides output quantity relative to the input quantity. The conventional detectable signal can be current, voltage, impedance, fluorescence, piezoelectricity, temperature, etc. [99]. The main transducer mechanisms, along with the analytes they are able to detect, are mentioned in Table 3.

There are two general categories of detection in the field of biosensors: label-based and label-free sensors. Label-based sensors include tag molecules or an dye indicators for detection [119,120]. Fluorescence, radioactivity and chemiluminescence represent key types of detection mechanisms for label-based sensors that have been widely used [121]. There are few potential drawbacks for label-based sensors. For instance, there is a complicated method to assure the label will not block any important active sites on tagged molecules, or the method leaves large amounts of contaminated reagents for radioactive labels. Less sensitivity in fluorescence sensor due to quenching is another problem.

**Figure 3:**
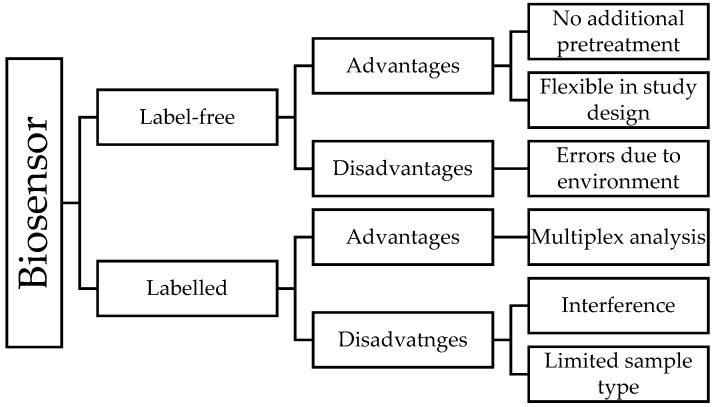
Biosensor categories, advantages and disadvantages [122,123,124].

In response to these problems, the rationale for the direct detection of analytes was initiated. Label-free sensors comprise a more novel technique, by which sensors can directly monitor the interaction in the testing media. In general, label-free sensor includes a transducer that is able to convert the physical properties of the analytes into a quantifiable signal. Each method has its merits and drawbacks, which are summarized in Figure 3. However, up until now, these two methods have been broadly comparable in different conditions, and neither was better or worse than the other [98]. 

Overall, it is difficult to detect biological analytes directly because of their physical properties, including size or mass. For this reason, bioscientists have used tags or labels for molecules detection [125]. However, due to the challenges related to label-based sensors, including cost, preparation time, etc., the use of label-free sensors has started to trend among many researchers and applications [120,122,125,126]. 

There are several important parameters that can be used to measure biosensor performance, including selectivity, limit of detection (LOD) or sensitivity, speed of detection, size of the sensor, stability and sample processing. Label-free sensors usually have minimal sample processing time and are mostly stable [88,122]. Table 4 summarized these parameters.

In most cases, early detection of very low concentrations of target analytes is essential for taking effective action; therefore, there is a demand for rapid, inexpensive and highly sensitive analytical tools for biosensor detection. There should be some level of accuracy and specificity of detection, as well [127,128].

## 4. Electrospun Label-Free Sensors

Label-Free sensors can study molecular interaction without the modification of molecules, and without interference or binding with other molecules. As there is no need to label, this method avoids radioactive labels, which makes it more safe and clean [129]. Label-free procedures have lately been attracting lots of attention as an alternative to label-based methods. This includes fewer operating steps and significantly reduces both cost and operation time [130]. 

Each sensor can react based on its transduction mechanism, as mentioned above. In this review, we followed the transducer mechanism for label-free division, as it is more popular. Electrochemical sensors were the first reported type of biosensors [131]. The basis for this method is the chemical modification of electrodes, such as a metal surface and carbon electrodes. In contrast, optical and more specifically amperometric sensors are based on refractive index changes and change of electromagnetic fields to change in the characteristics of light, respectively [125,132]. 

### 4.1. Electrochemical Sensors

Electrochemical sensors consist of a reference electrode and a sensing or working electrode. The measurement is based on the change detection in resistance on the surface of the electrode as a result of interaction with an analyte. This interaction could produce a measurable current (amperometric), a measurable potential or accumulation of a charge potential at the working electrode (potentiometric). It could also produce a measurable change in conductivity (conductometric) between electrodes [133] to be used in a non-destructive technique for reliable analysis of surface conditions at electrode surfaces such as EIS (electrochemical impedance spectroscopy) [134]. The presence of a higher amount of analyte on the biosensor surface increases the resistance of the layer, which can be used for analyte quantification [87]. 

The key challenge to assembling a reliable and label-free biosensor is to immobilize biomolecules inside the electrode layer, therefore having a large surface area and conductivity can increase the efficiency of sensors. Iridium oxide is an excellent candidate for label-free sensors due to its metal-like conductivity and very low resistance ~50 µΩ cm [135]. A “wire-in-tube” structure can be made by electrospinning this material followed by temperature-controlled annealing. This structure provides high surface area and can be coated with biomarker detectors (chemical compounds) [136].

This process not only improves the electron transfer and surface area of the nanowires, but also simultaneously provides a stable matrix for conjugation of biomolecules. The fabricated label-free sensors can detect AFP in a range of 0.05 to 150 ng/mL and a detection limit of 20 pg/mL. 

TEM imaging reveals that the average diameters of the inside wire and the whole nanofiber are ∼70 and 110 nm, respectively. The coated fibers have a linear response in the range of 10–190 mV/s in pH 7.4. These nano-wires have high reliability, with a standard deviation less than 5% and only a 14% drop in detection range after 15 days [136]. 

In another study, by Xu et al., a label-free electrochemiluminescent (ECL) immunoassay was fabricated to detect aflatoxin B1 (AFB1) using magnetic nanofibers. Here, an 8% solution of Ploymethylmethacrylate in *N*,*N*-Dimethylformamide (DMF) with 0.5 wt% Fe_3_O_4_ nanoparticles was mixed to fabricated Fe_3_O_4_-NFs rods though an electrospinning process. Furthermore, carbon nanohorns (CNHs) were dispersed in DMF and deposited over the electrospun fibers as the sensing element. Maximum performance was achieved with a 6% concentration of Fe_3_O_4_ and 3 mg/mL concentration of CNHs.

**Figure 4:**
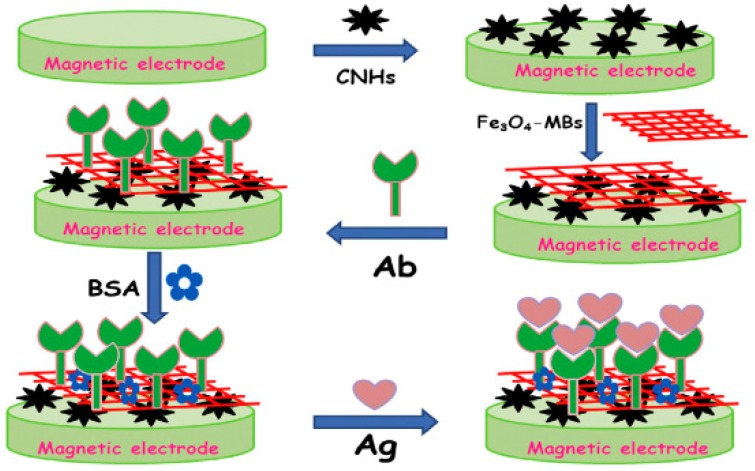
Schematic of the formation of sensing layer over the immunosensor using Bovine serum albumin (BSA), Aflatoxin B1 (Ab) and carbon nanohorns (CNHs), Magnetic beads (MBs) Fe_3_O_4_ [137]. Reprinted from the Sensor and Actuator B: Chemical, Vol 222, Guifang Xu, Shupei Zhang, Qingrong Zhang, Lingshan Gong, Hong Dai, and Yanyu Lin, Magnetic functionalized electrospun nanofibers for magnetically controlled ultrasensitive label-free electrochemiluminescent immune detection of aflatoxin B1, 707–713. Copyright (2016) with permission form Elsevier.

These magnetic nanofibers coated with CNHs were developed as a highly conductive, biocompatible, and high surface area material to detect ECL signals generated through the deposition of antibodies on the surface. The developed linear sensor has a range of 0.05 to 200 ng/mL and a limit of 0.02 ng/mL [137]. Figure 4 depicts the schematic of sensing layer formation on a magnetic electrode.

Electrochemical sensors have also been employed to detect DNA. While most DNA sensors use special instruments and well-trained operators, there is a huge demand for simpler and faster label-free biosensors for DNA detection. Label-free sensors can provide more accurate results in less time. Thus, the use of nanotechnology and nanoscale sensors can improve results and the performance of sensors [138]. 

As was reported by Tripathy et al., nano-sized biosensors have been desired for healthcare applications due to their low content detection, high sensitivity and miniature size. In this work, a semiconductor of manganese oxide was used in the shape of nanofibers to detect label-free DNA hybridization, with the limit of 120 e^−21^ M. To fabricate these nanofibers, 8% weight solution of PAN/DMF was mixed with Manganese (II) acetate tetrahydrate. After heat treatment, the solution was electrospun to make a porous membrane over an aluminum foil. The casted layer was then calcinated at 500 °C to make manganese oxide nanofibers. The diameter of the fabricated fibers was in the range of 100–300 nm for non-calcinated and 20–150 nm for calcinated fibers. DNA hybridization was detected using a glassy carbon electrode and cyclic voltammetry, electrochemical impedance spectroscopy, and differential pulse voltammetry. In all three methods, the hybridization of the DNA was observed, and this shows that both resistive or capacitive setups can be used [139]. 

**Figure 5:**
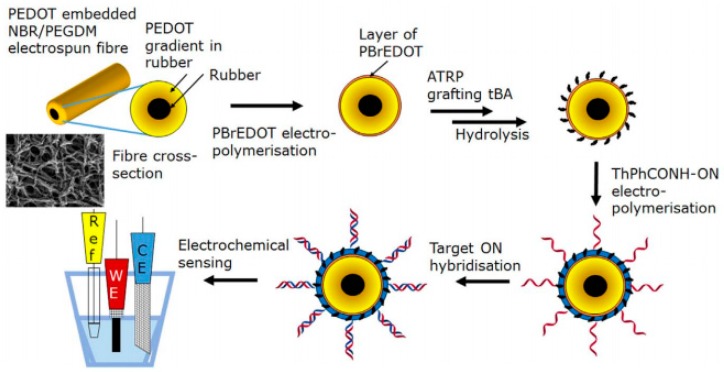
The fabrication of the PEDOT gene sensor using electrospun films [140]. Reprinted from The Biosensors and Bioelectronics, vol. 100, Thomas E. Kerr-Phillips, Nihan Aydemir, Eddie Wai Chi Chan, David Barkera, Jenny Malmströma, Cedric Plesse, and Jadranka Travas-Sejdic, Conducting electrospun fibres with polyanionic grafts as highly selective, label-free, electrochemical biosensor with a low detection limit for non-Hodgkin lymphoma gene, 549-555., Copyright (2018) with permission from Elsevier.

In another study, a label-free sensor based on electrochemical spectroscopy was developed. Here, a conductive fiber was produced by the electrospinning of NBR rubber, embedded with a conductive poly 3,4-ethylenedioxythiophene (PEDOT) matrix and poly acrylic acid (PAA) chains attached to the surface. The fibers were electrochemically polymerized with a DNA sensing layer made of 6,6-((2,5-di(thiophen-2-yl)-1,4-phenylene(bis(oxy))dihexanoic acid- Oligonucleotide (ThPhCONH-ON) and 2,2′-(2,5-bis(2-(2-(2-methoxyethoxy) ethoxy)ethoxy)-1,4-phenylene) dithiophene (ThPhEG) to detect mismatches in DNA sequences, with a limit of 1 aM. In this method sensors were highly selective to T–A mismatches and showed a detection range on the order of 10^−8^ [140]. The fabrication process is presented in Figure 5.

Hazardous material detection is another interesting area for label-free sensor applications. Supraja et al. developed a resistive-based label-free immunosensor to detect atrazine, a toxic chemical that attacks the human endocrine system. The developed fibers were able to detect concentrations of 10−21 g/mL, with a limit of 0.22 × 10^−21^ g/mL and a sensitivity of 52.54 (kΩ/μg·mL^−1^)/cm^2^. The fibers were made by electrospinning a 5% of manganese (III) acetate tetrahydrate solution mixed with 7% (w/w) of polyacrylonitrile and N, N-dimethylformamide. The spun fibers were then calcinated at 550 °C using a muffle furnace to form metal oxide fibers [139]. Later, biosensors were also made by immobilizing an anti-atrazine-antibody to the fiber’s surface; fibers were placed on a glassy carbon working electrode (GCE/MNF). Here, the surface of the GEC/MNF was functionalized using mercaptopropionic acid. Layers of Ethyl-3-(3-dimethylaminopropyl) carbodiimide (EDC) and N-hydroxysuccinimide (NHS) were deposited using layer-by-layer self-assembly to the surface of the MNFs, followed by incubation to stabilize the layers. The antibodies were attached to the GCE/MNF to enable atrazine detection by targeting the CO-NH bond of atrazine to the surface of fibers [139]. 

Electrochemical sensors made via electrospinning processes have also been made to detect hypoglycemia. Here, the base of these sensors is used as an agent to oxidize glucose and detect its level through the generated energy. While having higher surface area and smaller size sensors can improve efficacy, electrospinning methods have also been utilized to make glucose sensors using different metallic materials (e.g., copper, silver and gold) that are mixed with semiconductive materials (e.g., indium thin oxide and carbon nanofibers) that are imbedded in polymers such as poly(vinylidene fluoride). Recently, research in this area has focused on making smaller-sized and more aligned fibers in order to improve the detection rates of these glucose sensors [141]. 

#### Amperometric Sensors

Amperometric sensors continually measure current at a fixed potential, which is proportionally related to the concentration of the target analyte. This comes from the oxidation/reduction of an electroactive species in a biochemical reaction. Amperometric sensors are inexpensive and highly sensitive. They provide a wide linear signal range which is proportional to analyte concentration and can be extensively used in concentration measurements of different molecules in chemical analysis and environmental and biological detection [142].

**Figure 6:**
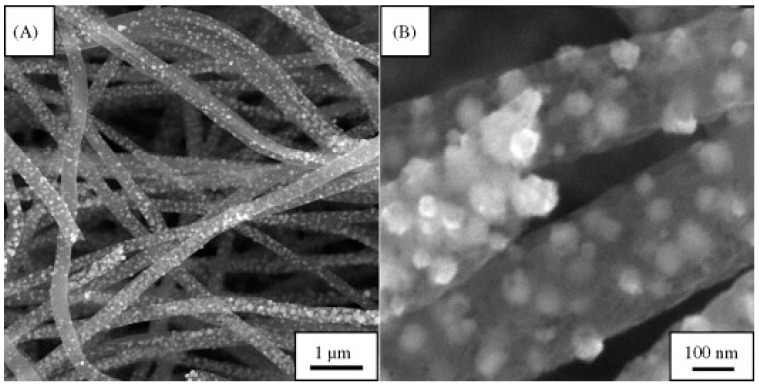
SEM images of the NiCF composite at (**A**) low and (**B**) high magnification [144]. Reprinted from the Analytica Chimica Acta, Vol 663, Yang Liu, Lei Zhang, Qiaohui Guo, Haoqing Hou, and Tianyan You, Enzyme-free ethanol sensor based on electrospun nickel nanoparticle-loaded carbon fiber paste electrode, 153–157, Copyright (2010) with permission form Elsevier.

Ethanol detection is very important for many applications, yet most ethanol sensors are based on immobilization provided by alcohol dehydrogenase or oxidase [143]. However, this method has side effects such chemical or thermal instability. The fabrication of enzyme-free sensors can lead to more precise and stable sensors. Several researches have been developed for enzyme-free alcohol detection methods using different nanoparticles. For example, Liu et al. [144] used nickel nanoparticles loaded in nanosized carbon fiber to acts as an ethanol sensor. The nanofibers were made by mixing polyacrylonitrile and nickel acetylacetonate (NiAA) in a DMF solution, followed by electrospinning. The fibers were then treated in different temperatures to be carbonized and completely stabilized. The fibers then were deposited over copper wires to make electrodes. The diameter of the fabricated fibers was in the range of 200 to 400 nm, and the fibers were 10 microns in the length, while the embedded nickel nano particles were in range of 50 nm and were observed within the structure (Figure 6) [144]. This method is simpler and more stable compared to the previously reported method of making the carbon fibers first and then coating them with a layer of nickel [145].

With these materials, the detection of ethanol is based on the redox of Ni(II)/Ni(III) in voltages of 0.6 and 0.43 V by formation of a NiOOH layer on the surface of the fibers. The detection limit of these stable sensors was found to be 0.25 mM and the calibration curve was linear up to 87.5 mM, with a standard deviation of only 4.1%. These sensors have higher amperometric response compared to bulk nickel sensors due to their efficiency and smaller size. In addition, the sensor is very stable in a desiccator, with only a 3% drop after one month and a standard deviation of 3.8%; the sensors can also be renewed by polishing. These sensors have lower detection limits and increased stability compared to other types of sensors.

Laccase biosensors have been developed by Fu et al. based on using electrospun carbon nanofibers and copper/carbon nanofibers to detect catechol. To make the Cu/CNF fibers sensors, polyacrylonitrile was mixed with dimethylformamide and a polyvinylpyrrolidone aqueous solution with Cu(Ac)_2_. The solution was then electrospun and pre-oxidized at 280 °C, followed by carbonization at 900 °C. The fabricated fibers were then mixed with lactase and deposited over a glassy carbon electrode to make the sensor. A sensitivity of 33 uA/mM and a range between 9.95 × 10^−6^ to 9.76 × 10^−3^ M and detection limit of 1.18 μM was achieved using Cu/CNFs/Lac/Nafion/GCE sensors [146].

### 4.2. Chemiresistive Sensors

Numerous chemiresistive sensors have been made using electrospinning methods to achieve easy-to-fabricate micron-sized sensors with high sensitivity, increased detection rates, stability, and cross sensitivity [147,148,149]. For example, Prakash et al. created the aligned SU-8 photoresist infused with functionalized multiwall carbon nanotubes (MWCNT) through electrospinning. This material was used to detect biomarkers such as myoglobin. In this work, adding MWCNTs to aligned photoresist nanofibers improved both sensing and conductivity. The main goal of this research was to consider the ultrasensitivity of these fibers and the ideal MWCNT content in the fibers [150].

Electrospinning was used to deposit MWCNT SU-8 nanofibers (Micro Chem, Newton, MA, USA) on a Cu- glass wafer. The copper microelectrode array for this nano-biosensor was developed by deposition of copper ribbons over coated glass substrate using lithography technique. The stripes of copper, with a thickness of 200 µm and the gap of 50 µm, were deposited by sputtering copper. For fiber fabrication, MWCNT (with diameters of 5–20 nm and lengths of 1–10 µm) and SU-8 2015 (a mild viscosity epoxy) were mixed through mild probe sonication in chloroform solution. The maximum weight of MWCNT in epoxy was 8%. This mixture was deposited over the microelectrode array by electrospinning. Aligned polymer/nanomaterial in the form of nanofibers provides conductivity and biocompatibility, while by functionalizing the nanomaterials, biosensing could also be achieved [150]. 

**Figure 7:**
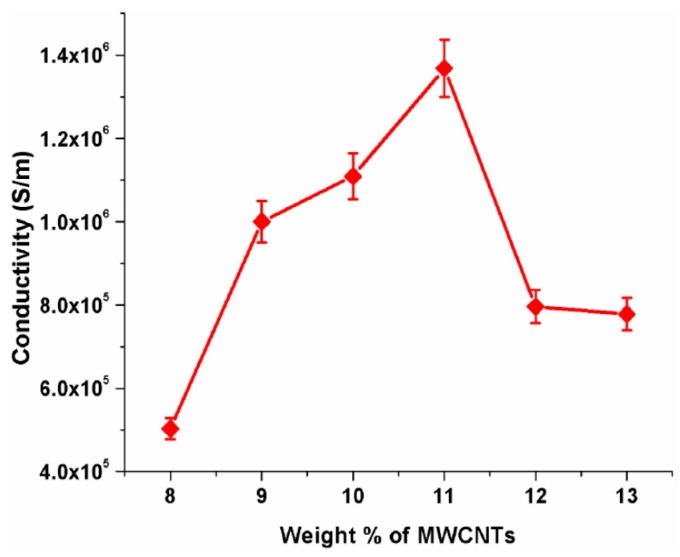
Electrical conductivity change by MWCNT content [150]. Reprinted from the MDPI Sensors, Vol 16, Matta Durga Prakash, Siva Rama Krishna Vanjari, Chandra Shekhar Sharma, and Shiv Govind Singh, Ultrasensitive, label Free, chemiresistive nanobiosensor using multiwalled carbon nanotubes embedded electrospun SU-8 nanofibers, 1354., Copyright (2016) with permission form MDPI.

The results revealed that the diameter of the electrospun fibers was ~280 ± 28 nm, and that the MWCNTs were imbedded in the polymer structure. Interestingly, the conductivity of these fibers is not linearly related to MWCNT content, as shown in Figure 7. By increasing the MWCNT wt%, the conductivity will increase and then decline by adding more MWCNT. This is due to the interconnection of parallel MWCNT conductive paths, increasing the total impedance of the network. The testing of myoglobin also reveals that the functionalized nanofibers can detect the myo-antigen more precisely with no memory effect, with a detection range of fg/mL to µg/mL [150].

Although nanowire sensors are in wide use due to their small size and high aspect ratio, their fabrication remains a challenge. Electrospinning can be a solution to fabricate small-diameter (sub 100 nm) carbon nanowire sensors. Carbon Microelectromechanical Systems (C-MEMS) can be developed using photolithography to provide different sensor patterns, and structures; they can also be integrated into microfluidics or lab on a chip device resulting in label-free biomaterial sensors [151]. 

As was reported by Thiha et al., carbon nanowires have been made through the carbonization of electrospun photoresist. Here, SU-8 photoresist was electrospun over a silicon substrate with a thickness of 100 nm and then carbonized through annealing. The resulting nanowires were then functionalized by activation of the carboxylic bonds. The carboxylic functionalized carbon nanowires were then used to develop a chemiresistive sensor to detect biomarkers in small batch (5 µL) samples of Salmonella bacteria; a detection limit of 10 CFU/mL and a turnaround 5 min was demonstrated. Detection was based on the change in resistance between carbon nanowires in the presence of the bacteria. As was reported, by attaching the bacteria to the nanowires, the resistance of the sensor dropped linearly based on the concentration of bacteria. This strategy can also be used in other biosensor applications such as DNA extraction, or fast lab on a chip bacteria detectors [151].

Electrospinning is widely used in making semiconducting nanostructured metal oxides to detect gases with high sensitivity and selectivity. Nanostructured semiconductive metal oxides such as titanium dioxide, zinc oxide, tungsten oxide and tin oxide have been used to detect precise concentrations of chemicals in the range of parts per million [152]. In another study by Kim et al., resistive-based TiO_2_/poly (vinyl acetate) nanowires were developed through electrospinning over platinum electrode arrays. This material was then used to detect nitrogen dioxide with a limit of 1 ppb and 90% recovery time of 8.4 ± 0.5 min after 10 min of exposure (at a rate of 500 ppb). 

**Figure 8:**
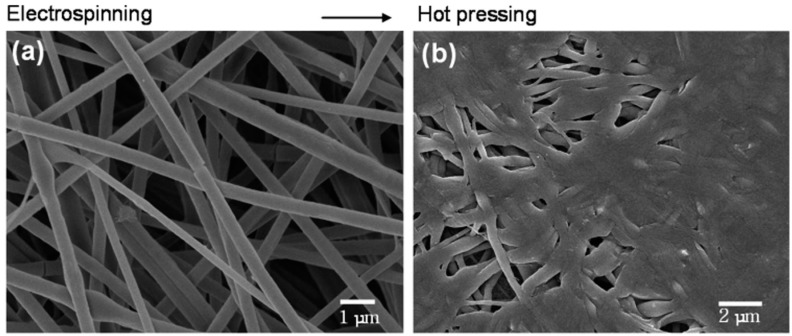
(**a**) SEM image of the as-spun TiO_2_/PVAc composite fibers fabricated by electrospinning from a DMF solution. (**b**) SEM image of TiO_2_/PVAc composite fibers after hot pressing at 120 °C for 10 min [143]. Reprinted from the Nano Letters, Vol. 6, Il-Doo Kim, Avner Rothschild, Byong Hong Lee, Dong Young Kim, Seong Mu Jo, and Harry L. Tuller, Ultrasensitive, ultrasensitive chemiresistors based on electrospun TiO_2_ nanofibers, 2009–2013., Copyright (2006) with permission form American Chemical Society.

The sensing mechanism of these fabricated sensors is based on an N to P inversion of the semiconductive material in the presence of NO_2_, which results in a reduction of total resistance. Here, to increase sensitivity, nanofibers with a thickness of 200–500 nm (Figure 8a) were compressed over a platinum electrode (Figure 8b) to form a single sheet with high porosity for maximum gas accessibility. This study also showed that the specific surface area of the non-pressed and pressed fiber films were 31.22 m^2^/g and 138.23 m^2^/g respectively, which explains the significant improvement in the sensitivity of pressed sensors [153].

### 4.3. Optical Sensors

Optical biosensors are one of the most common classes of biosensors. Optical detection is performed by interaction of a biorecognition element with the optical field. The basic principle here is based on the use of a photodetector to measure the change in optical properties such as absorption, reflectance, emission, or interferometric pattern in the presence of an analyte [154]. In a label-free optical sensor, the detected signal is generated directly by the interaction of the target analyte with the transducer. Surface Plasmon Resonance (SPR), evanescent wave fluorescence, optical waveguide interferometry, colorimetric and fluorescent are different types of optical sensors [155]. 

There is limited research being conducted on the optical properties or applications of neat electrospun nanofibers, as most of the polymers do not exhibit optical properties. However, one could modify the polymer before electrospinning by including doping nanofibers or other components to incorporate optical properties [156]. 

**Figure 9:**
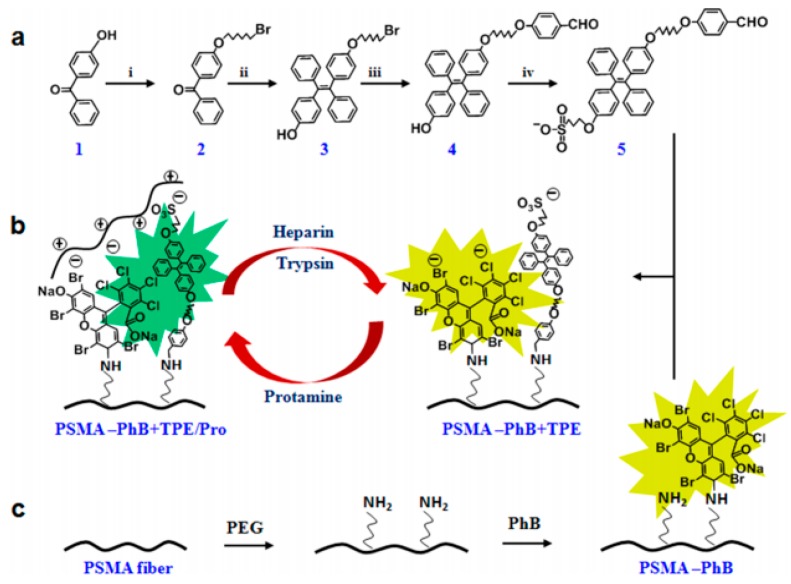
Synthetic rout of TPE and the principles of the change of color for electrospun PSMA fliers: (**a**) Synthetic route of TPE, (**b**) schematic of color changing process due to protamine adsorption and interaction of TPE and PhB, and (**c**) formation of the grafted PhB on fibers [157]. Reprinted from the ACS Applied Materials and Interfaces, Vol. 9, Long Zhao, Tao Wang, Qiang Wu, Yuan Liu, Zhoujiang Chen, and Xiaohong Li, Fluorescent Strips of Electrospun Fibers for Ratiometric Sensing of Serum Heparin and Urine Trypsin, 3400–3410., Copyright (2018) with permission from ACS.

In a study performed by Zhao et al., a fibrous strip was developed to detect the level of trypsin, up to 8 µg/mL, for pancreas transplant patients. Here, a layer of tetraphenylethene (TPE) with phloxine B, as a protamine absorbent layer, was deposited over electrospun PSMA fibers (using Poly ethylene glycol to make the strips). By introducing trypsin to the strips, protamine is removed to reveal the fluorescence of phloxine B at 574 nm and the emission of the TPE derivative at 472 nm. This causes a significant and visible color change throughout the fibers, which is illustrated in Figure 9. Here, the presence of trypsin changes the color of the grafted polybrominated biphenyl (PbB) layer over the fibers [157].

With Cu^2+^ ions generated in an array of biological reactions, the efficient and effective detection of these particles is desired. Nitrogen-doped carbon dots can be used to identify label-free Cu^2+^ through fluorescent sensing. 

Li et al. [158] conducted research on the Nitrogen-doped carbon dots (N-CD) that were fabricated by the breakdown of electrospun carbon nanofibers (CNFs). The CNFs were fabricated by electrospinning polyacrylonitrile (PAN). The electrospun fibers were then added to sulfuric and nitric acids, sonicated for 2 h, and stirred overnight. Later, the solution was neutralized by adding NH_4_OH and was dialyzed to make N-CD. The N-CDs could then be used to detect Cu^2+^ thought the interaction of N and O groups of the N-CDs. The results showed a concentration range of 0 to 10 µM and a limit of 5 nM with high selectivity.

Another method of making optical sensors by electrospinning is to use PVA. A sensor was made using Ceria nanoparticles and PVA to detect metal cations such as Ce^3+^. The Ceria nanoparticles were added to the PVA matrix to make cross-linked Ceria-PVA fibers for the purpose of electrospinning. The fibers could detect Ce^3+^ ions in the presence of a peroxide solution suitable for environmental monitoring [159]. 

#### 4.3.1. Surface-Enhanced Raman Scattering (SERS)

**Figure 10:**
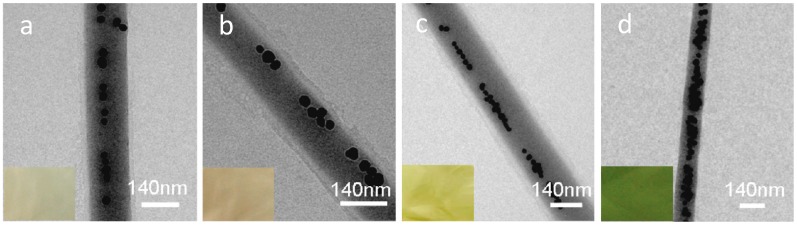
Typical TEM image of Ag/PVA nanofibers with the molar ratio of PVA/Ag (**a**) 530:1, (**b**) 530:2, (**c**) 530:3, and (**d**) 530:4. [159]. Reprinted from the ACS Nano, Vol 3, Xianfeng Wang, Dian He, Bo HuQiao-Feng, YaoKan Wang, and Shu-Hong Yu, Large-Scale synthesis of flexible free-Standing SERS substrates with high sensitivity: Electrospun PVA nanofibers embedded with controlled alignment of silver nanoparticles, 3993–4002, Copyright (2009) with permission from American Chemical Society.

Surface-enhanced Raman scattering (SERS) is a modification to the Raman spectrum that provides a higher detection range for vibrational signals compared to conventional Raman spectroscopy. Additionally, improving electromagnetic filed and chemical reactions can enhance SERS results [160]. One key technique for doing this involves the use of nanomaterials instead of metallic substrates for SERS. Silver nanoparticles are a good candidate to be used in SERS substrates, as they can be impregnated into polymers or nanolayers [161]. A surface-enhanced Raman scattering (SERS) substrate was successfully developed by He et al. [160] using an electrospinning method where chain-like arrays of silver nanoparticles were imbedded in PVA nanofibers. The developed fibers could be used in SERS detection of 4-mercaptobenzoic acid molecules and demonstrated enhancement factors up to 109, reproducibility, stability and low concentration detection (10^−6^ M). In this research, authors employed microwave-synthesized Ag nanoparticles with PVA solution to fabricate Ag/PVA nano fibers. The uniformly mixed DMF-based solution with Ag nanoparticles was then extruded via electrospinning to produce green-colored nanofibers. As the PVA/Ag solution was colorless, the green color signaled Ag aggregates. The thickness of these fibers was reported to be ~170 nm, and their length was limited to several millimeters. This three-dimensional structure, made by randomly displaced fibers, has a uniform surface and a high aspect ratio. Figure 10 shows TEM images for different Ag/PVA molar ratios and corresponding mat color that resulted.

**Figure 11:**
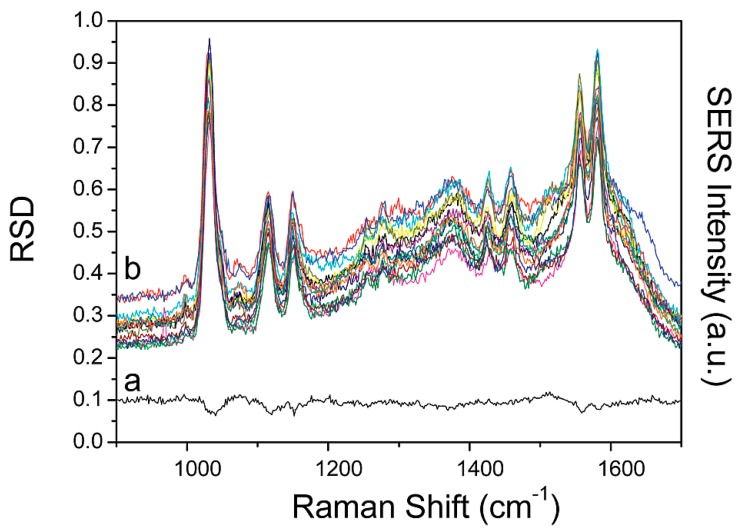
RSD-SERS graph. (**a**) RSD value curve of SERS of 10^−6^ M 4-MBA collected on the randomly selected 15 places on the Ag/PVA nanofiber mat. (**b**) Series of SERS spectra collected from the randomly selected 15 places on the Ag/PVA nanofiber mat [160]. Reprinted from the ACS Nano, Vol 3, Xianfeng Wang, Dian He, Bo HuQiao-Feng, YaoKan Wang, and Shu-Hong Yu, Large-Scale synthesis of flexible free-Standing SERS substrates with high sensitivity: Electrospun PVA nanofibers embedded with controlled alignment of silver nanoparticles, 3993--4002., Copyright (2009) with permission from American Chemical Society.

Researchers have claimed that the presence of Ag nanoparticles increased the local electromagnetism, which in turn leads to enhanced molecular sensing with high sensitivity. The molecule absorption was tested by adding 4-(MBA) molecules to the substrate. The results revealed that the 530:3 molar fibers had the maximum spectra, and the short chain structure provided the best enhancement. In addition, the thickness of the Ag/PVA did not play a key role in enhancing the results. The developed substrate showed great repeatability in the detection of 4-MBA molecules, with a concentration limit of 10^−6^ M, which is quite low, with a major Raman peak of less than 0.07 for the relative standard deviation (RSD) curve of 15 SERS spectra and a shelf life of more than a month (Figure 11) [159]. 

This method can be used to detect very low concentrations of target molecules, or even single molecules. As SERS is very reproducible and portable, this substrate can be considered to be a chemical sensor or biosensor [162]. 

#### 4.3.2. Colorimetric Sensors 

**Figure 12:**
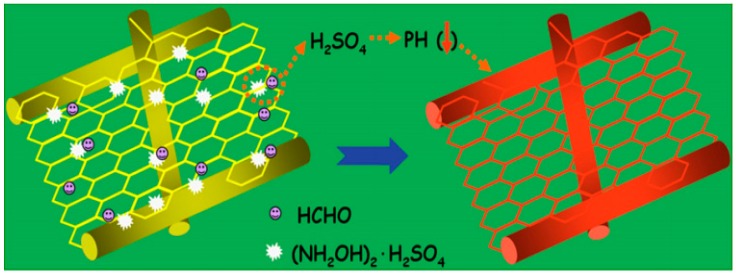
Illustration of the colorimetric detection of formaldehyde based on the nylon 6 nano-fiber/nets (NFN) membrane [165]. Reprinted from the Sensors and Actuators B: Chemical, Vol. 163, Xianfeng Wang, Yang Si, Jialin Wang, Bin Ding, Jianyong Yu, and Salem S. Al-Deyab, A facile and highly sensitive colorimetric sensor for the detection of formaldehyde based on electro-spinning/netting nano-fiber/nets, 187 (2012) with permission from Elsevier.

The colorimetric sensor principle is based on the change in reflectance at a specific wavelength in the presence of a specific chemical. Recently, multiple methods have been developed to increase the efficiency of these sensors using nano materials and nano fabrication [163,164]. Wang et al. developed a highly sensitive colorimetric sensor through the electrospinning of methyl yellow-impregnated nylon 6 nanofibers. This sensor, designed to detect formaldehyde, provides a color change from yellow to red in the presence its presence, with a detection limit of 50 ppb. Here, the nylon 6 was dissolved in formic acid and then electrospun to form a highly porous film consisting of nanowires with the two different diameters, one of 18 ± 4 nm and one of 150–250 nm; these represented the sensing and supporting wires, respectively. The fabricated film was later coated with a sensing layer of hydroxylamine sulfate and methyl yellow aqueous solution. By exposing the formaldehyde to this coated film, a low energy reduction band at the wavelength of 550 nm could be detected. This change is due to the reaction of formaldehyde with sulfuric acid trapped in the network structure of the nylon nano fibers [165]. The detection mechanism is illustrated in Figure 12.

As reported by Yew et al., a novel reaction membrane based on polycaprolactone nanofibers (PCL) has also been used as a biosensor for lateral flow assays. Here, PCL nanofibers were functionalized with hydroxyl and carboxyl chains by adding NaOH to achieve maximum efficacy and detection. In addition, having the transition chains on the PCL surface can increase the selectivity of the sensors to detect specific proteins. The developed sensor can detect ssDNA with a detection limit of 0.5 nM [166]. Another use of these sensors is to develop paper-based sensors for medical, food safety and environmental monitoring. While some polymers are hydrophobic, deposition of the fibers over a hydrophilic substrate can improve sensitivity significantly. By deposition of a PCL layer over a hydrophilic nitrocellulose membrane, better adsorption is possible, and higher sensitivity can be achieved. This method was also used to develop a sensor for detecting bacteria and other biological compounds [167].

#### 4.3.3. Fluorescence Sensors

As the name indicates, fluorescence sensors work based on fluorescence emission. When the sensor is hit by a photon, it can absorb the energy of that photon and reach the excited state. With relaxation time, molecules release a fluorescence emission [168]. When using this method, the intensity can be read directly, and there is no need for a reference. There is no need to carry out the covalent labeling of fluorophores in label-free fluorescent sensors, making this sensor more capable compared to the label ones [169,170].

Conjugated polymers made by electrospinning are widely used in the fabrication of highly sensitive fluorescent sensors. There is a huge selection of transparent fibers made by electrospinning that are suitable for optical applications, such as polymethylmethacrylate (PMMA), poly acrylic acid (PAA), and polyamide fibers [29]. 

Zhang et al. [113] reported fabrication of a highly sensitive fluorescent glucose biosensor by the electrospinning method using graphene quantum dots/PVA. Graphene quantum dots (GQDs) were synthesized by the hydrothermal method. PVA was added to the solution, followed by ultrasonication. The homogeneous PVA/GQD solution was used for electrospinning to fabricate nanofibrous membrane. The voltage and distance were fixed at 15 kV and 12 cm, respectively. This electrospun membrane was tested for glucose fluorescence detection. The porous structure of nanofibers generates excellent media to absorb and penetrate H_2_O_2_. Hydrogen peroxide can impact the fluorescence intensity of GQDs through the formation of surface oxide traps for electrons. The results revealed the good reproducibility and long-term stability of the fabricated sensor, with a linear range of 0.25–24 mM and 10 μM as the limit of detection for glucose.

poly(phenylenevinylene)/polymethylmethacrylate (PPV/PMMA) conjugate fibers have been made through electrospinning, in order to detect metallic cations such as Cu^2+^ and Fe^3+^ in different biological and environmental systems [110]. In another effort, a bio compatible fluorescent sensor for detecting Cu^2+^ was developed using photoluminescent polymer nanodots, made by the hydrothermal method from grass [171]. 

As was reported by Wang et al., novel electrospun fibers have been made by attaching poly pyrene methanol (PM) to PAA through covalent bonding to detect Fe^3+^ Hg^2+^ and 2,4-dinitrotoluene. Here, the fibers of PM-PAA were fabricated by electrospinning of PM-PAA with cross-linkable polyurethane latex mixture, followed by a 225 °C curing for crosslinking the membrane. The developed membrane could be used to detect many metal cations with low concentration, for instance, 1.1 × 10^6^ (M^−1^), 8.9 × 10^5^ (M^−1^) for Fe^3+^, Hg^2+^, respectively. The detection limit is 2–3 orders of magnitude greater than PM-PAA film, which can be attributed to the higher surface area of the electrospun membranes [172]. 

## 5. Challenges and Future Scope

While substantial advancement has been made in the electrospinning field, there are still several areas in which further improvement is required. Complexity of fabrication, limit of spinnable materials, uniformity of fibers, and repeatability are some of the limitations and challenges. The fact that, in electrospinning, electrospun nanofibers are mostly randomly oriented has been limiting in terms of the repeatability of the final structures. This limitation has been improved through the use of new electrospinning methods such as coaxial, which gives better control over electrospun nanofiber orientation.

There are many challenges in the fabrication of sensors using electrospinning. The main one is to make a uniform and spinnable mixture. In this case, the uniformity of the mixture and the ratio of the materials are crucial [173]. Absorption of the sensing material and the lifetime of the fibers is becoming increasingly important. While many of these sensing layers are deposited using layer-by-layer self-assembly, having a uniform zeta potential over the fiber structure is highly desirable. In addition, polymer scaffolds should not react with the sensing material or the environment [139,174].

In addition, the ultra-fine size, surface area, flexibility, porosity and reproducibility of the electrospun fibers are important in sensing applications [174]. Having a larger surface area is a key to having higher sensitivity. Therefore, reductions in fiber diameter lead to higher surface areas. Other than that, creating 1D and 2D structures increases the surface area of the produced fibers [147,175,176].

Furthermore, the ability to fabricate portable sensors without the need for bulky instrumentation is one of the current challenges that researchers are hoping to overcome in the future. The ability to detect very low concentrations, and the addition of new nanomaterials to fabricate more sophisticated sensors through surface modification, are two other key directions in this area [177].

## 6. Conclusions

Electrospinning is simple, low-cost, and effective, and is the most powerful method for fabricating diverse nanostructures. Current advances in this technology by developing new electrospinning methods such as coaxial, modified coaxial and multiple nozzles raise the possibility of creating desired fibers even from unspinnable materials.

Electrospun fibers are employed in a broad range of applications, from biomedical to sensory. High porosity, large surface area, and ease of surface modification make electrospun fibers a great candidate for producing highly sensitive and very selective label-free sensors. 

This review provides a short overview of electrospun nanofibers applications, with an emphasis on biosensor applications. With respect to this area, focus is placed on label-free sensors, pertaining to both recent advances and fundamental research. We introduced the main types of nanofiber-based label sensors with electrochemical, amperometric, chemoresistive, optical, SERS and colorimetric sensors, classified by the transducer mechanisms. Current challenges in this area and prospective future work were also discussed. Moving forward, electrospun label-free sensors could be a new horizon for fast and accurate sensors in the sensory world.

## Figures and Tables

**Table 1:** Effect of electrospinning parameters on filament formation [2,16].

**Polymer**	Higher Molecular Weight	Smaller Deposition Area, Larger Fibers
	Lower molecular weight	Larger deposition area, smaller fibers, bead formation
**Viscosity**	High	Larger fibers, spinning prevention
	Low	Discontinuation of filament formation, beads formation
**Humidity**	High	Spraying instead of electrospinning, wet fiber formation,
	Low	Broken filaments, nozzle clogging
**Temperature**	High	Less viscosity and lower fiber dimensions, uniform formation of fibers
	Low	High viscosity and larger fiber dimensions, nozzle clogging

**Table 2:** Comparison study of spinnable polymers based on solvent and viscosity.

Polymer	Solvent	Molecular Weight	Wt% of Polymer	Viscosity (cps)	Reference(s)
*PEO	Water	400,000	1–4%	100–2000	[17]
**PEO**	DMF	300,000	7%	1480	[18]
**PEO**	Chitosan/water 1:1 weight	600,000	2%	3000	[19]
*PVA	Water	124,000–186,000	12%	2591	[20]
**PVA**	Ethanol/water 1:1 weight	78,000	8–10%	900–3000	[21]
*PVP	Ethanol	1,300,000	4.50%	3450	[22]
**PVP**	Water	360,000	10%	3480	[23]
**PVP**	DMF	360,000	14%	4439	[24]

* poly ethylene oxide (PEO), Polyvinyl alcohol (PVA), Poly vinyl pyrrolidone (PVP).

**Table 3:** Transduction methods used in biosensors [95].

Transducer	Example	Analyte(s)	References
Electrochemical	Potentiometric: ion-selective electrodes, ion-selective field effect transistors	Bacteria	[100,101]
		Disease	[102]
	Amperometric: Clark oxygen electrode, solid electrolyte gas sensors, electronic noses	Bacteria	[103,104,105]
		Sugar	[106,107]
Optical	Optical fibers, surface plasmon resonance, total internal reflection fluorescence, absorbance, luminescence	Bacteria	[108,109]
		Heavy metals	[110,111,112]
		Sugar	[113]
High frequency	Piezoelectric crystals, surface acoustic wave sensors	Disease	[114,115]
		Protein	[116]
Heat sensitive	Calorimetric sensors	Gases	[117,118]
Miscellaneous	Whole cells, single molecules, carbohydrates, conducting polymers		

**Table 4:** Effective parameters in biosensor performance.

Parameter	Explanation
Sensitivity	Smallest amount of target molecules can be detected
Selectivity	Sensor responds only to target molecules
Speed	Fast detection with no sacrifice in accuracy
Size	Compact, portable device
